# Enhanced production of recombinant proteins in *Corynebacterium glutamicum* by constructing a bicistronic gene expression system

**DOI:** 10.1186/s12934-020-01370-9

**Published:** 2020-05-26

**Authors:** Manman Sun, Xiong Gao, Zihao Zhao, An Li, Yali Wang, Yankun Yang, Xiuxia Liu, Zhonghu Bai

**Affiliations:** 1 National Engineering Laboratory of Cereal Fermentation Technology, Jiangnan University, Wuxi, 214112 China; 2 Key Laboratory of Industrial Biotechnology, Ministry of Education, School of Biotechnology, Jiangnan University, Wuxi, 214122 China; 3 Division of Life Science and Center for Chinese Medicine, Hong Kong University of Science and Technology, Hong Kong, China; 4 Jiangsu Provincial Research Center for Bioactive Product Processing Technology, Jiangnan University, Wuxi, 214122 China

**Keywords:** *Corynebacterium glutamicum*, Recombinant protein, BCD expression system, Screening fore-cistron sequences, PΙNP

## Abstract

**Background:**

*Corynebacterium glutamicum* is a traditional food-grade industrial microorganism, in which an efficient endotoxin-free recombinant protein expression factory is under developing in recent years. However, the intrinsic disadvantage of low recombinant protein expression level is still difficult to be solved. Here, according to the bacteria-specific polycistronic feature that multiple proteins can be translated in one mRNA, efforts have been made to insert a leading peptide gene upstream of target genes as an expression enhancer, and it is found that this can remarkably improve the expression level of proteins under the control of inducible tac promoter in *C. glutamicum.*

**Results:**

In this research, the *Escherichia coli* (*E. coli*) tac promoter combined with 24 different fore-cistron sequences were constructed in a bicistronic manner in *C. glutamicum.* Three strong bicistronic expression vectors were isolated and exhibited high efficiency under different culture conditions. The compatibility of these bicistronic vectors was further validated using six model proteins- aldehyde dehydrogenase (ALDH), alcohol dehydrogenase (ADH), RamA (regulator of acetate metabolism), Bovine interferon-α (BoIFN-α), glycoprotein D protein (gD) of infectious bovine rhinotracheitis virus (IBRV) and procollagen type Ι N-terminal peptide (PΙNP). All examined proteins were highly expressed compared with the original vector with tac promoter. Large-scale production of PΙNP was also performed in fed-batch cultivation, and the highest PΙNP production level was 1.2 g/L.

**Conclusion:**

In this study, the strength of the inducible tac promoter for *C. glutamicum* was improved by screening and inserting fore-cistron sequences in front of the target genes. Those vectors with bicistronic expression patterns have strong compatibility for expressing various heterogeneous proteins in high yield. This new strategy could be used to further improve the performance of inducible promoters, achieving double competence of inducible control and high yield.

## Introduction

*Corynebacterium glutamicum* has been used as an important industrial microorganism to produce amino acid since the 1950s, during which people have gained abundant knowledge about its growing condition and cellular mechanism [1–3]. Trials of developing a recombinant protein expression system in this microorganism have been made in recent years given its characteristics of endotoxin-free, low extracellular protease abundance and the ability of protein secretion [4]. Many industrial enzymes and polypeptides for medical uses such as single-chain variable fragment (scFv) [5] and N‑terminal pro‑brain natriuretic peptide (NT-proBNP) [6] have been successfully produced in *C. glutamicum*. However, compared with the most widely used host *Escherichia coli* (*E. coli*), the application of *C. glutamicum* for industrial protein production is in its infancy because of some drawbacks, e.g. lower protein yield [5, 7], lower transformation efficiency [5] and limited genetic tools [6].

To enhance the protein yield in *C. glutamicum* expression system, various strategies including screening promoters and other genetic parts, optimizing culture conditions and engineering host cells [6, 8, 9] have been carried out. Among them, manipulation on promoter element is regarded as the most straightforward way since promoter confers direct control of the transcription initiation process and can explain up to 80% variance of corresponding protein level. Relevant works mainly focused on the development of promoter libraries from endogenous promoters [10–13], promoter mutants [14] and synthetic promoters [2, 15, 16], However, owing to the strong expression strength and low background level, the tac promoter (P_tac_) from *E. coli* is still the most widely used promoter in *C. glutamicum* for its inducible traits and high efficiency.

Gene expression levels are not always consistent with promoter strength because of the effects of other genetic elements such as the 5′untranslated region (5′UTR) and translation initiation region (TIR) on mRNA stability and mRNA secondary structure [17]. When an unfavorable secondary structure happens in mRNA transcript, little or no expression of protein was detected in the general monocistronic expression cassette (promoter-5′UTR-single target gene) although the target gene was under the control of a strong promoter. Solutions by optimizing the TIR/UTR sequence for each protein target is very tedious and time-consuming [18–20]. To build a vector with good compatibility for various proteins, a bicistronic design (BCD) expression cassette (promoter-5′UTR-leading peptide gene-target gene) can be considered [21]. In this expression system, a short peptide coding sequence was inserted upstream of the target gene to facilitate its expression by translation coupling [21]. This is increasingly attractive to recombinant protein expression in recent years. Two possible reasons have been proposed to explain the observation of the improvement of expression by using such bicistronic promoter: (1) An efficient translation of the first cistron (also termed “fore-cistron”) prevents the formation of a stable mRNA secondary structure at the beginning of the second cistron (target protein sequence). (2) Two coding sequences (CDS) have translational coupling effect: ribosome herein initiates the second translation process right after it falls from the stop codon of the first cistron—usually a sequence-economic and easily translated polypeptide [22]. Although the mechanism and influencing factors of the bicistronic expression system have not been fully elucidated so far, there are still some successful expression cases using bicistronic expression patterns in *E. coli* [21, 23, 24] and lactic acid bacteria [19]. Our previous works [25, 26] also demonstrated that the expression level and translation efficiency of the endogenous bicistronic promoters could be stronger than the corresponding monocistronic promoters for gene expression in *C. glutamicum*. However, previous studies in *C. glutamicum* simply took the endogenous monocistronic promoters together with its following native N-terminal coding sequence (fore-cistron) as bicistronic promoters and some of which did not show enhancement of protein expression compared with the monocistronic one [26]. It seems that the arbitrary introduction of a fore-cistron may not confer a remarkable improvement of the promoter strength. Given the complexity of the whole expression frame, it is possible that those bicistronic promoters may perform better if coordinating with a proper fore-cistron sequence, and the embedded “fore-cistron elements” could be a further enhancer for protein expression under the control of a strong promoter. Therefore, we intend to combine P_tac_ with different fore-cistron sequences to further enhance this widely used inducible promoter in *C. glutamicum*.

In the present study, we built the P_tac_ into a bicistronic manner for enhanced and stable gene expression in *C. glutamicum* CGMCC1.15647. First, we evaluated the performance of 24 fore-cistron sequences in a bicistronic promoter pattern on the expression of the reporter Enhanced Green Fluorescent Protein (EGFP). Next, we selected the top three strongest bicistronic vectors for further study of expression compatibility and translation efficiency. Using the selected bicistronic vectors, the enhanced production of recombinant proteins in *C. glutamicum* was successfully demonstrated using six protein models-aldehyde dehydrogenase (ALDH), alcohol dehydrogenase (ADH), RamA (regulator of acetate metabolism), Bovine interferon-α (BoIFN-α), glycoprotein D protein (gD) of infectious bovine rhinotracheitis virus (IBRV) and procollagen type Ι N-terminal peptide (PΙNP). Large-scale production of PΙNP was also performed in fed-batch cultivation.

## Results

### Construction of bicistronic P_tac_ expression system for enhanced recombinant proteins expression

**Fig. 1:**
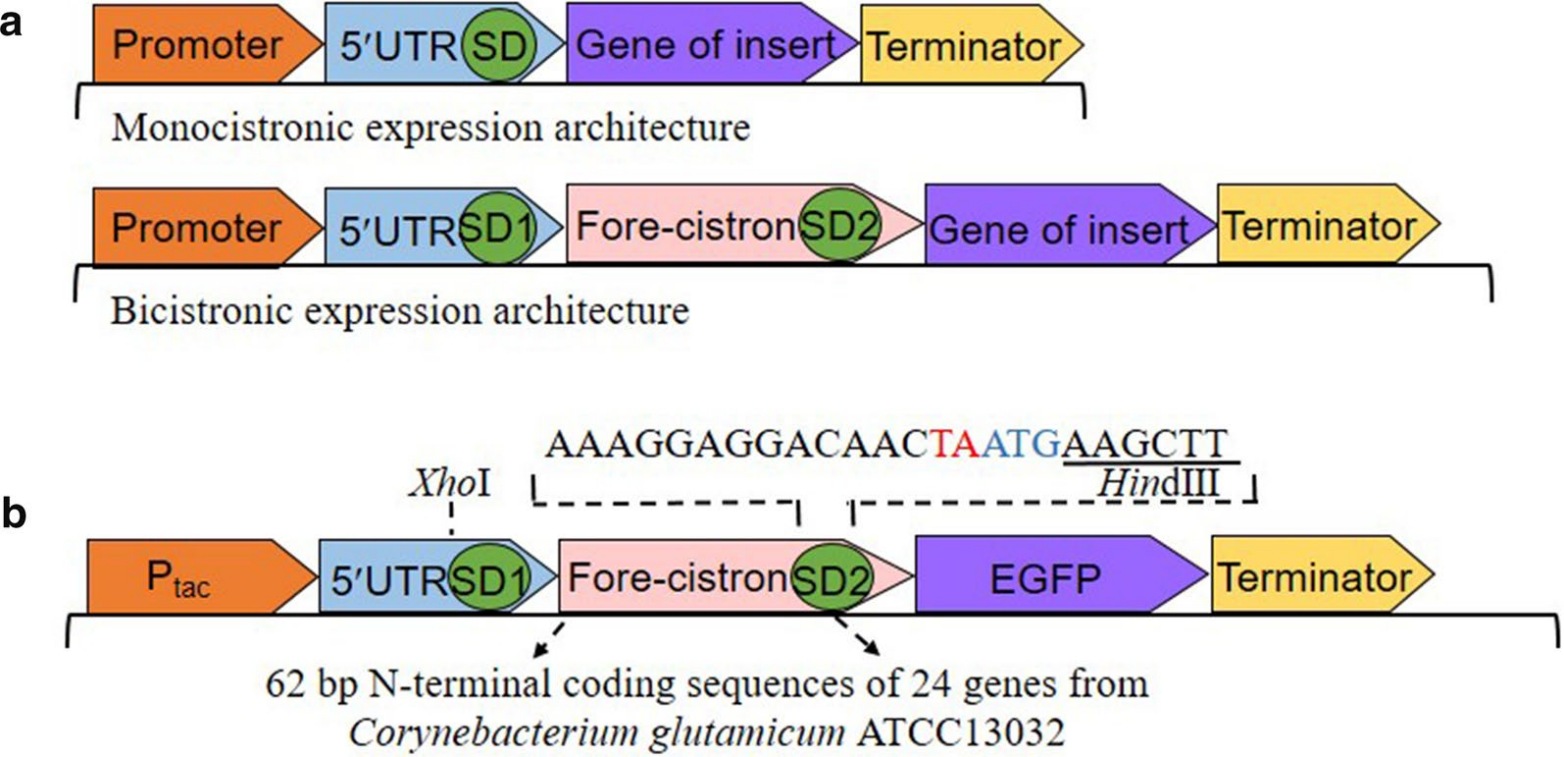
Bicistronic expression structure. **a** Monocistronic and bicistronic expression structure, a bicistronic expression structure including a strong promoter, 5′UTR with its conserved SD sequence (SD1), the fore-cistron sequence, the second SD sequence (SD2) and the target gene. **b** The structure of BCD expression plasmids. The 62 bp of the N-terminal coding sequences of 24 genes were constructed into the pXMJ19-EGFP according to this bicistronic expression model and followed a conserved SD2 sequence AAAGGAGGACAACTA

Appropriate fore-cistron can effectively improve the effect of the endogenous promoters when constructed in a bicistronic pattern [4, 25]. To enhance recombinant protein expression in *C. glutamicum*, we constructed a bicistronic P_tac_. The N-terminal 62 bp of the open reading frame (ORF) from 24 genes were taken as the fore-cistron sequences embedded between P_tac_ and target gene, these 24 genes were originated from 12 highly transcribed genes (GEO accession number: GSE77502) and 12 highly expressed genes (provided by Schaffer S) in *C. glutamicum* [25, 26] (Additional file 1: Table S3). The highly transcribed genes were ranked according to transcriptomic data, and the highly expressed genes were ranked based on protein abundance in proteomic data. To compare the variance of expression, the EGFP reporter system was constructed under the control of these bicistronic promoters. The bicistronic expression structure was shown in Fig. 1. Since there is no RBS sequence in the original plasmid pXMJ19, a conserved Shine-Dalgarno sequence (SD1) AAAGGAGGACAACC was added at the N-terminal of fore-cistron sequence to initiate its translation. According to the characteristics of the bicistronic expression structure, a second conserved SD sequence (SD2) was introduced at the C-terminal of the fore-cistron sequence and it was designed as a translation coupling sequence AAAGGAGGACAACTAATG (Fig. 1b). This structure enables the protein synthesis to stop after completing the synthesis of the first-cistron (62 bp N-terminal CDS) and then re-initiate the translation of the target gene (EGFP). *LacI* gene upstream of the P_tac_ promoter confers its inducible trait, so the expression of target protein requires the induction of IPTG.

### Characterization of effects of different fore-cistron sequences on expression of EGFP

**Fig. 2:**
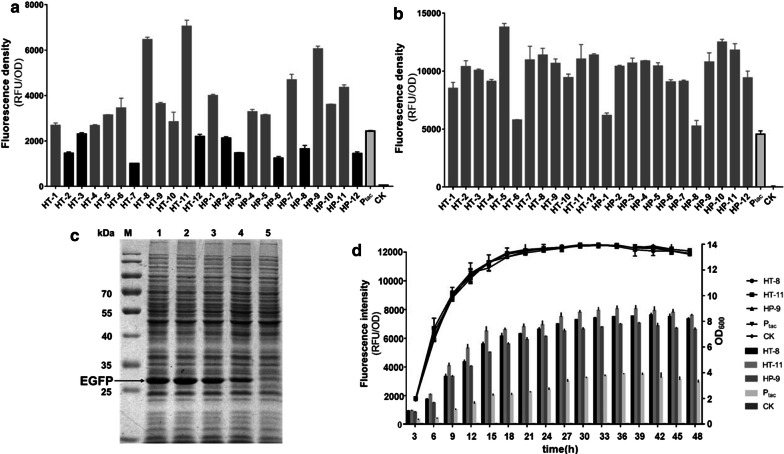
EGFP expression level of the BCD expression vectors. pXMJ19-EGFP and pXMJ19-0 were used as control and control check (CK). **a** The fluorescence intensity of BCD expression vectors contained different fore-cistron sequences in *C. glutamicum*. **b** The fluorescence intensity of enhanced BCD expression vectors in *E. coli*. **c** The SDS-PAGE analysis of EGFP expression of the top three BCD expression vectors. Lane M: Protein Marker 26616; lane1-3: pbtac-HT-8-EGFP, pbtac-HT-11-EGFP, pbtac-HP-9-EGFP; lane4: pXMJ19-EGFP; lane5: CK, pXMJ19 without the EGFP gene. **d** The fluorescence intensity and growth curve of the top three strongest BCD expression vectors at different time points

After successfully constructed in *E. coli* DH5α, each plasmid was transformed into *C. glutamicum* CGMCC1.15647, which was previously proved a better host for recombinant protein expression [8]. To characterize the effects of different fore-cistrons on EGFP expression, the fluorescence intensity was measured and normalized to OD_600_ after 24 h flask cultivation. The control here was P_tac_ alone without the insertion of fore-cistron. The HP (highly expressed genes in *C. glutamicum*) and HT (highly transcribed genes in *C. glutamicum*) sets represented two different sequence sources of fore-cistron as described above. The results showed that the expression intensities were varying among different BCD vectors (Fig. 2a), with 15 of 24 constructs having higher EGFP expression levels than the P_tac_ plasmid. The EGFP expression level of the top three strongest vectors (pbtac-HT-8, pbtac-HT-11, and pbtac-HP-9) were 2.65-, 2.89- and 2.48-fold of the monocistronic control, respectively. The SDS-PAGE analysis matched well with fluorescent intensity measurement (Fig. 2c). Next, we examined the fluorescence intensity of these three EGFP expression constructs at different time points. The expression levels of those three simultaneously peaked at 39 h with similar expression increasing tendency, which is about 12 h later than the respective OD_600_ values attained at the stationary phase (Fig. 2d). Interestingly, unlike in *C. glutamicum*, in *E. coli* all the 24 BCD expression vectors showed higher EGFP fluorescence intensity than the P_tac_ control (Fig. 2b). The top three strongest vectors described above exhibited similar expression ability and a bit weaker than the highest pbtac-HT-5. These results indicated that the fore-cistron sequences have varied degrees of enhancement for P_tac_ in *C. glutamicum*. Screening an appropriate fore-cistron sequence for bicistronic promoter constructing is particularly important in *C. glutamicum*.

### Comparison of the strength of BCD expression vectors under different culture conditions

Medium composition and growing condition could affect physiological characteristics and gene expression profiles [27], altering the expression efficiency of foreign genes. Promoter strength, which is a direct influence factor for protein expression, may also be altered sometimes. Besides, the inserted fore-cistron sequences were originated from endogenous genes, which may confer additional expression characteristics to the P_tac_ under certain culture conditions. Taken together, the expression stability of the selected bicistronic P_tac_ should be examined.

**Fig. 3:**
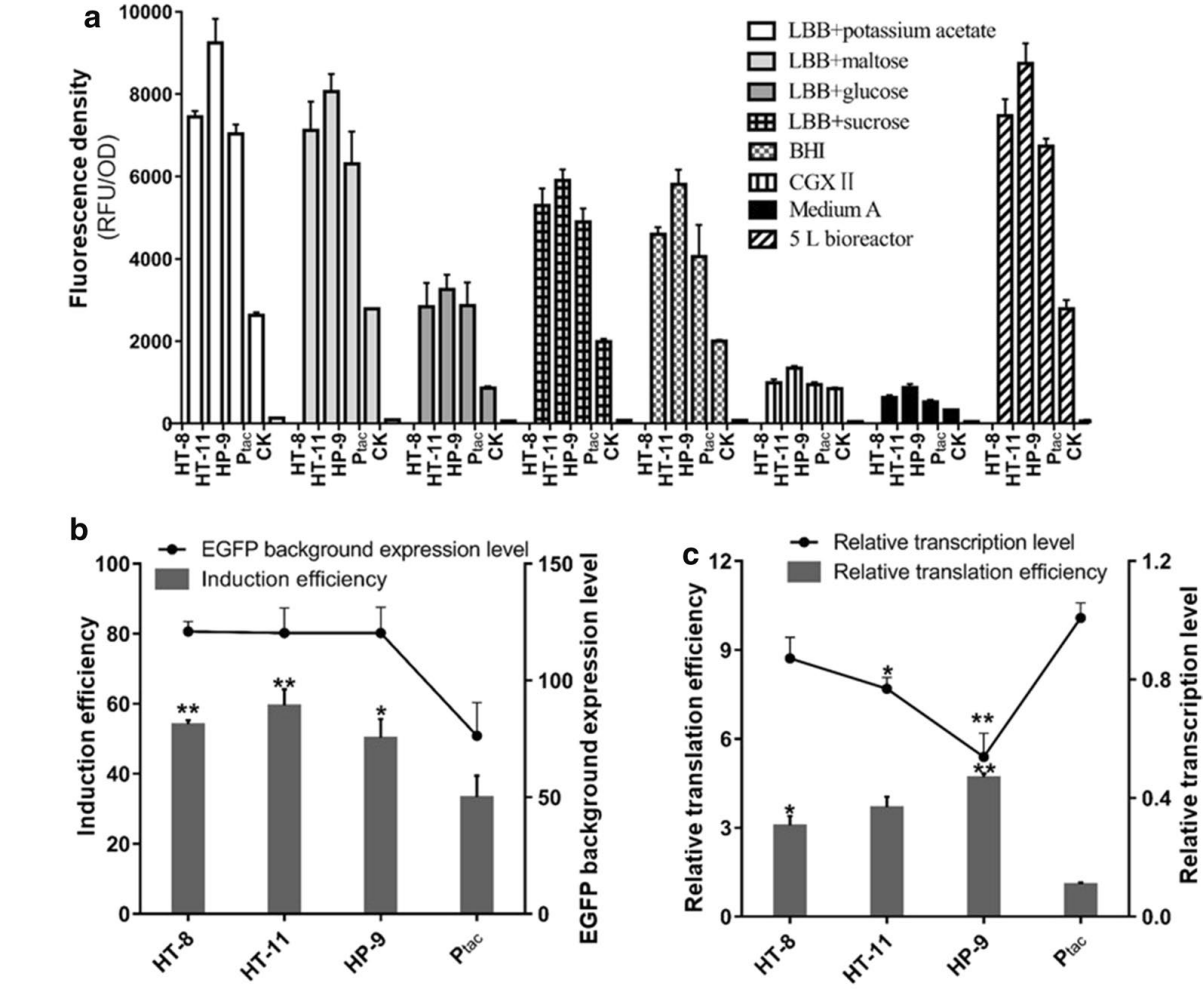
The expression stability and translation efficiency of the top three strongest enhanced expression vectors. Fluorescence intensity was normalized to OD_600_ of each construct. The asterisks indicate a significant difference between BCD expression vector and the original vector containing a P_tac_ at *P < 0.05 and **P < 0.01 using the t-test. **a** Fluorescence intensity of pbtac-HT-8, pbtac-HT-11 and pbtac-HP-9 under different culture conditions. **b** The EGFP background expression level and induction efficiency of the top three strongest vectors (*P < 0.05, **P < 0.01). **c** Relative transcriptional level and relative translation efficiency of EGFP for each enhanced expression vector (*P < 0.05, **P < 0.01)

LBB medium is supplemented with different carbon sources (potassium acetate, maltose, glucose, and sucrose, with a final concentration of 10 g/L). LBB medium together with the previously reported CGXII, medium A and BHI media were recruited here. Strains were inoculated in 10 mL of above 7 media and cultivated for 24 h. As showed in Fig. 3a, the fluorescence intensity varied significantly in different media, while the three bicistronic expression vectors still exhibited higher EGFP fluorescence intensities compared with the monocistronic P_tac_ control. The differences of expression in other media were also similar to that in LBB and the vector pbtac-HT-11 was still the strongest one. Moreover, to testify the stability of these vectors under fed-batch cultivation, we employed a 5 L bioreactor and found that the trend of expression was also consistent with the flask tests (Fig. 3a). These results indicated that the selected three BCD expression vectors exhibited good stability and higher strength in different culture media and in large-scale cultivation.

### Comparison of induction efficiency and translation efficiency of BCD expression vectors

Although the selected bicistronic vectors showed higher EGFP intensity, the background expression levels of these three vectors with different fore-cistron sequences still needs to be examined. We measured the induction efficiency and background expression level of these enhanced BCD vectors. Results showed that the top three strongest bicistronic expression vectors maintained a comparable low-level background expression and strong induction efficiency (Fig. 3b). To further assess the influence of bicistronic P_tac_ on EGFP expression, we quantified the mRNA levels of EGFP by qRT-PCR and calculated the translation efficiency. The transcription level and translation efficiency of P_tac_ control here are defined as 1. Compared to the P_tac_, all three vectors showed lower transcription levels, while their translation efficiency was significantly improved (P < 0.05) (Fig. 3c). Among them, vector pbtac-HP-9 had the highest translation efficiency (4.61 times higher than the P_tac_ control, P < 0.01). These results indicated that the screened three bicistronic vectors showed higher induction efficiency and translation efficiency although their transcriptional levels were not improved. Therefore, introducing a proper fore-cistron sequence results in a higher translation efficiency of the second cistron (target gene) under the control of P_tac_.

### Enhanced expression of ALDH, ADH, RamA, BoIFN-α, gD and PΙNP

**Fig. 4:**
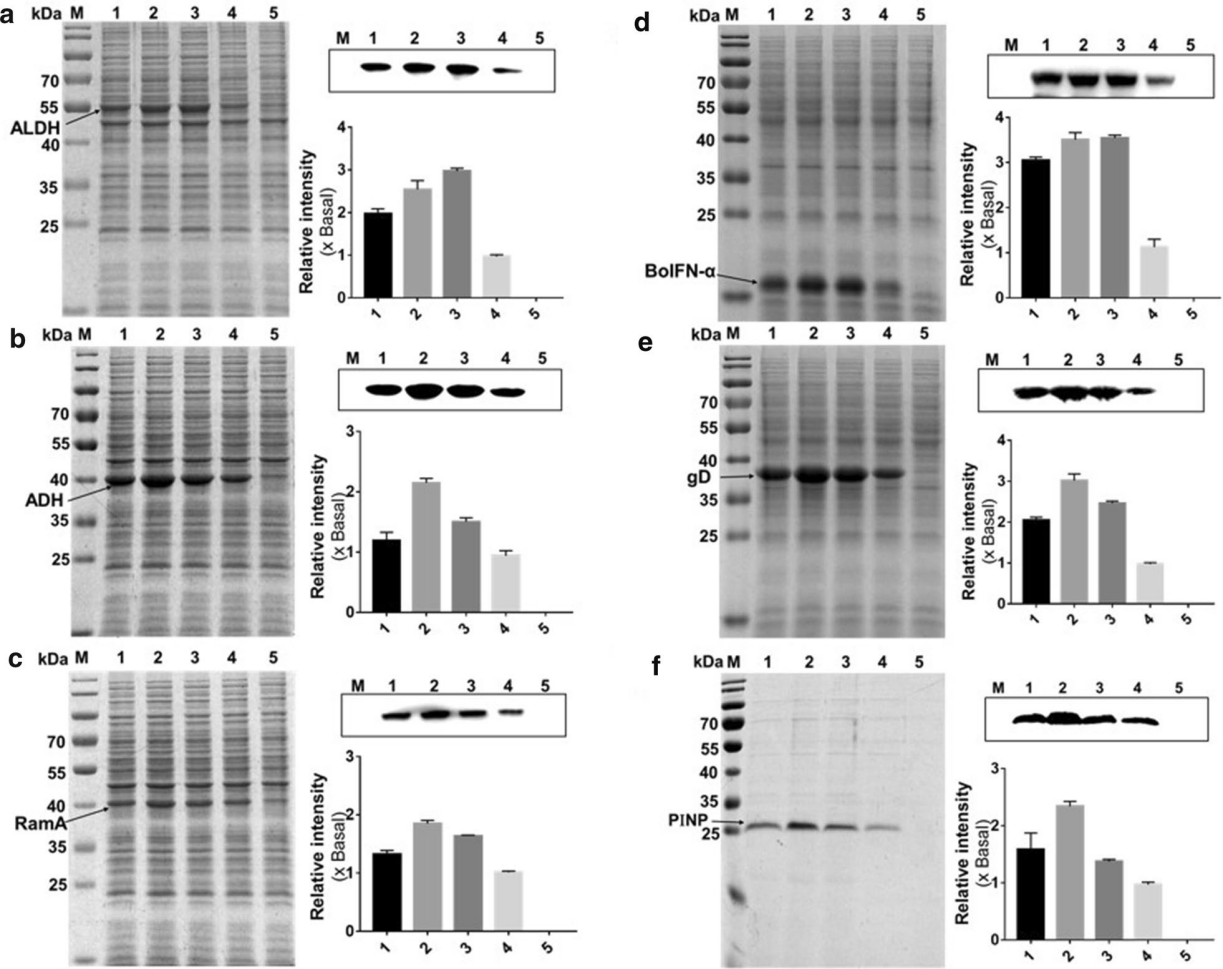
Protein expression levels of the top three strongest BCD expression vectors. pXMJ19-ALDH, pXMJ19-ADH, pXMJ19-RamA, pXMJ19-BoIFN-α, pXMJ19-gD, pXMJ19-PΙNP were used as control, pXMJ19-0 were used as CK. Arrow represents the target protein. Lane M: Protein Marker; lanes 1–3 represented expression vectors: pbtac-HT-8, pbtac-HT-11and pbtac-HP-9; lane 4: control, protein expression with a monocistronic P_tac_ promoter; lane 5: CK, pXMJ19 without exogenous protein genes. **a** SDS-PAGE and western blotting analysis of ALDH. **b** SDS-PAGE and western blot analysis of ADH. **c** SDS-PAGE and western blotting analysis of RamA. **d** SDS-PAGE and western blotting analysis of BoIFN-α. **e** SDS-PAGE and western blotting analysis of gD. **f** SDS-PAGE and western blotting analysis of PΙNP

Introducing fore-cistron sequences upstream of different recombinant protein coding-sequences will form different mRNA transcripts, and may alter expression property [28]. To explore whether the strength of three bicistronic P_tac_ could be influenced by the variance of target genes and evaluate the performance of these vectors on the expression of other recombinant proteins, we applied these three well-performed vectors to the expression of several other valuable proteins. All proteins have a 6× histidine tag at the C-terminal and were tested by western blotting assay using a monoclonal horseradish peroxidase (HRP)-conjugated anti-His_6_ antibody. The strength of protein bands was quantified using Image J software. We first substituted EGFP with three endogenous proteins-ALDH, ADH, and RamA in the constructs of pbtac-HT-8, pbtac-HT-11, pbtac-HP-9, and pXMJ19, respectively. ALDH, ADH, and RamA are important functional proteins in ethanol metabolism and involved in ethanol oxidation [28–30]. After 24 h of cultivation, the production yield of these proteins was determined by SDS-PAGE analysis and western blotting. As showed in Fig. 4a–c, all the proteins were successfully expressed and can be seen on SDS-PAGE. The proteins yield of these three BCD expression vectors were generally higher than the P_tac_ control.

To further evaluate the ability of these vectors in the production of foreign proteins, we also employed these BCD vectors to examine and produce three exogenous proteins-BoIFN-α, gD, and PΙNP. BoIFN-α and gD play important roles in IBR prevention; BoIFN-α is a cytokine with a broad-spectrum antiviral activity that prevents bovine infectious diseases [31]; gD protein is the main immunogenic protein of infectious bovine rhinotracheitis virus (IBRV) [32]. The BoIFN-α and gD expression levels in different vectors were shown in Fig. 4d, e. Compared with the P_tac_, all three BCD expression vectors had higher expression levels of BoIFN-α and gD. Vector pbtac-HT-11 had the highest expression level of BoIFN-α and gD which were 3.62- and 3.13-fold higher than the P_tac_ control. PΙNP is a useful marker for bone formation activity and bone disorders [33]. The measurement of PΙNP content is recommended as an aid in diagnosing patients with Osteoporosis. Thus, the production of PΙNP in *C. glutamicum* provides a potential for diagnostic use. The *PΙNP* gene with the cspB signal peptide was amplified and ligated to the above expression vectors. The PΙNP expression levels of the three enhanced vectors were shown in Fig. 4f, all three BCD vectors showed higher production, and the pbtac-HT-11 was the strongest one with 2.34 times PΙNP yield of the control. These results showed that all six proteins were successfully expressed in *C. glutamicum*, and five proteins existed in soluble form except BoIFN-α. All three selected vectors exhibited better compatibility for expressing various proteins than the original monocistronic P_tac_.

### High-level production of PΙNP by fed-batch cultivation

**Fig. 5:**
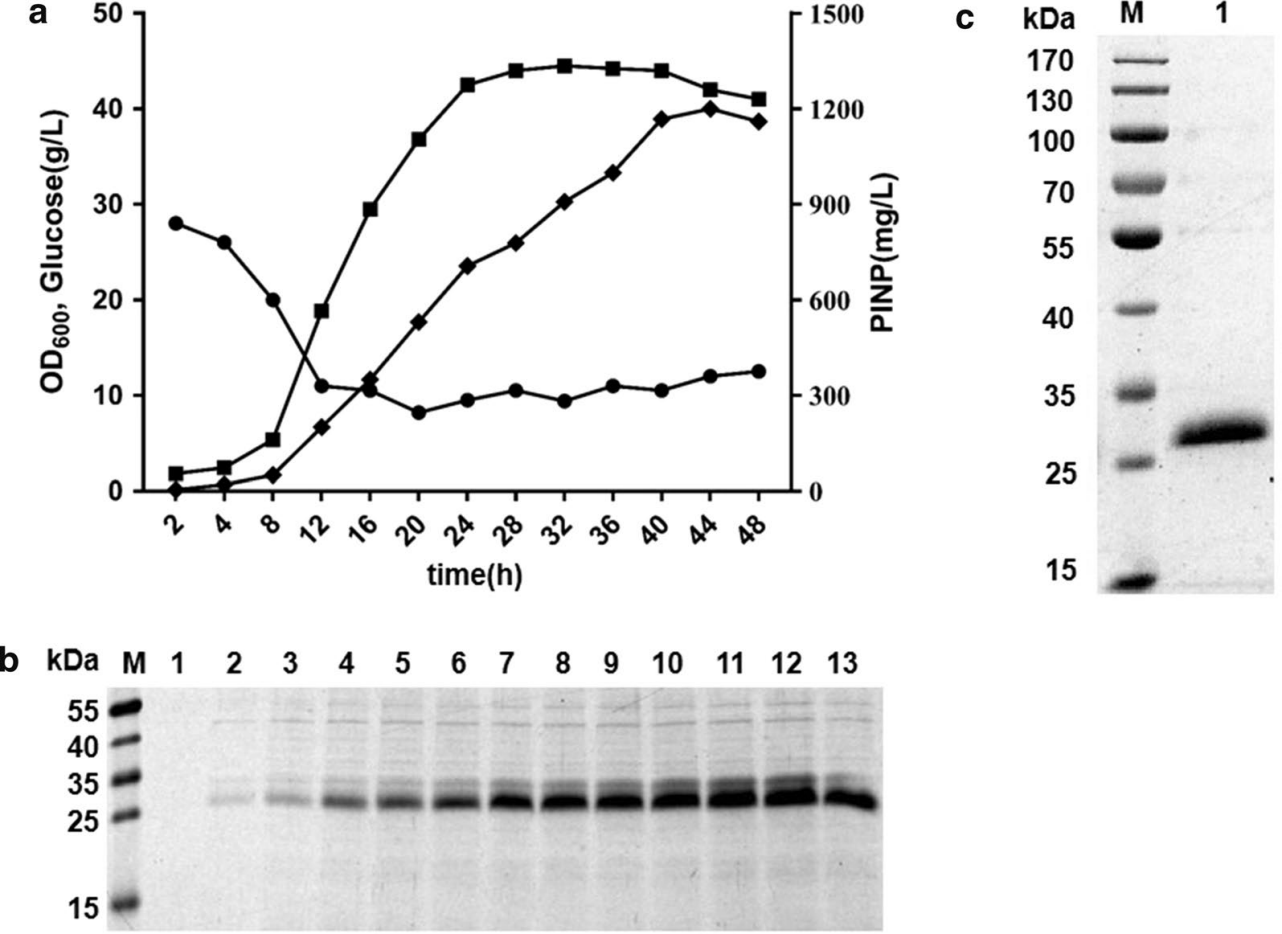
Fed-batch cultivation of *C. glutamicum* for the production of PΙNP. **a** Time profiles of cell growth (square), glucose concentration (circle), and PΙNP concentration (diamond) in the culture supernatant. **b** SDS-PAGE analysis of the culture supernatant. Lane M: Protein Marker; lanes 1–13; samples taken at 2, 4, 8, 12, 16, 20, 24, 28, 32, 36, 40, 44 and 48 h, respectively. **c** SDS-PAGE analysis of purified PΙNP. Lane M: Protein Marker; lane 1: purified PΙNP

To achieve high-level production of PΙNP, fed-batch cultivation with pbtac-HT-11 was carried out in a 5 L bioreactor system. The OD_600_ of the bacteria reached 44.5 at 32 h and then the cell density began to decrease gradually (Fig. 5a). We also measured the expression of recombinant protein at different time points by SDS-PAGE analysis. It could be seen that a clear PΙNP band firstly appeared at 4 h after inoculation, and the yield increased quickly in the stationary phases during cultivation (Fig. 5b). The PΙNP was no longer accumulated after 44 h cultivation, and the maximum yield in the culture supernatant reached 1.2 g/L by the Elisa kit assay (Rochecobas). The PΙNP expressed by pbtac-HT-11 was purified by a HisTrap HP affinity column. PΙNP was successfully purified with high purity (> 90%) after simple purification steps (Fig. 5c).

## Discussion

*Corynebacterium glutamicum* has been used as a microbial cell factory to produce various types of amino acids and organic acids for a long time [3]. In recent years, the well-established industrial facilities for *C. glutamicum* were being utilized to produce more valuable products including enzymes and biopharmaceuticals [5, 34]. Currently, much more efforts have been put into the development of *C. glutamicum* being used as a new host cell for recombinant protein expression, and new expression vectors [8, 25, 26], strong constitutive promoters have been discovered for supporting *C. glutamicum* to express protein with increased yield [5, 10]. However, a well-controlled process of recombinant protein production will be helpful to achieve satisfied productivity and also reduce manufacture costs. This is why P_tac_ is still the primary efficient inducible promoter widely used in *C. glutamicum* [35]. A new strategy for the development of expression vectors for *C. glutamicum* is optimizing the P_tac_ vector by adding some expression enhancers to the vector.

Bacteria have a special polycistron expression pattern in which several genes (cistrons) are transcribed into one RNA and translated separately. This enables us to adjust the expression level of one cistron by adding another cistron upstream of that. Target gene in a proper bicistronic system could have higher protein expression levels [19, 23]. However, simply connecting heterologous promoters to the coding sequence of the target protein may not efficiently initiate protein expression due to the formation of an unfavorable mRNA structure. In our previous studies, we took fragments which contain a promoter, 5′UTR and N-terminal coding sequence from heterogenous highly expressed genes, and connected it to the target protein [25, 26]. This may not help the expression either because a truncated N terminal coding sequence may not be a good delivery to efficiently send ribosomes to our target coding region. They were neither inducible genetic parts. This study developed an endogenous fore-cistron sequence screening strategy, we tested different fore-cistron sequences as an enhancer of the expression for the expression of the second cistron in *C. glutamicum*. The strongest three bicistronic promoters shown in our study remained higher potency than the control when expressing different proteins, so we inferred that the fore-cistron sequence could be developed as an independent expression enhancer. The reasons for the improved expression ability of these BCD vectors may be as follows: (1) The efficient translation of post-cistron depends on the prior translation of the fore-cistron. If the fore-cistron is well translated in the BCD expression structure, the translation of downstream gene may also be facilitated [25]. (2) BCD expression system prevents the formation of a stable mRNA secondary structure of the target protein sequence and then promotes the initiation of translation. Furthermore more elements, such as the length of the first cistron [36] and its amino acid composition [37] need to be investigated to evaluate the improved ability in combination with different promoters, UTR, SD sequences [19, 38], target genes, and different hosts—the designing principles established in *E. coli* may not be fully applicable to *C. glutamicum*, as the 24 promoters in our study showed a completely different intensity trend between the two hosts.

In *C. glutamicum*, various carbon sources are metabolized through different metabolic pathways, carbon sources and culture conditions were also reported to affect bacterial growth and protein expression [27, 39]. Since fore-cistron sequences were derived from the heterologous genes, they may confer other expression characteristics to the bicistronic system under certain culture conditions. Thus we explored the stability of the BCD expression vectors contained different fore-cistron sequences and found all these vectors stably functioned under different culture conditions. We believe that these bicistronic vectors could better satisfy the needs of protein expression in *C. glutamicum*. We analyzed these screened vectors in expressing veterinary vaccines. IBRV is a serious pathogen of cattle and causes significant economic losses to the cattle industry. BoIFN-α and gD are the main vaccine protein for the prevention and treatment of IBR. Although there are precedents for BoIFN-α and gD expression [32, 40], the development is still in the laboratory stage. In this study gD and BoIFN-α were first highly expressed in *C. glutamicum* using the top three strongest bicistronic vectors, which provided a new host for industrial expression gD and BoIFN-α and provides a chance for *C. glutamicum* in the production of veterinary vaccine proteins. We also employed these enhanced BCD vectors to achieve high-level expression of PΙNP, an important biomarker and diagnostic protein in bone metabolism. To the best of our knowledge, this is the first report on the production of PΙNP in *C. glutamicum*. These results demonstrated strong compatibility of those bicistronic expression vectors and provide another potential use for *C. glutamicum* bicistronic expression system in the production of diagnostic proteins.

## Conclusion

In conclusion, a bicistronic expression system suitable for enhanced production of recombinant proteins in *C. glutamicum* was successfully constructed. To serve the purpose of a reliable target gene expression, the fore-cistron sequences screening strategy was adopted, and the usefulness of the BCD system for enhanced gene expression was also successfully demonstrated with six protein models including ALDH, ADH, RamA, BoIFN-α, gD and PΙNP. The highest PΙNP secretion level reached was 1.2 g/L. This is the first report on the production of PΙNP in *C. glutamicum.* The results of the present study suggest that these BCD vectors could better meet the requirements of enhanced production of recombinant proteins and overexpression of key enzymes in *C. glutamicum*.

## Materials and methods

### Strains and culture conditions

The bacterial strains and plasmids used in this study were listed in Additional file 1: Table S1, except for all the bicistronic expression vectors. *C. glutamicum* CGMCC1.15647, *C. glutamicum* ATCC13032, *E. coli* DH5α were stored in our laboratory. *E. coli* was grown in LB medium or on LB plates containing 1.5% (wt/vol) agar at 37 °C. Unless otherwise indicated, *C. glutamate* was cultured in LBB broth (LB + 10 g/L brain heart infusion, pH 7.0) at 30 °C for 24 h. The medium for the transformation of *C. glutamate* was LBHIS medium (5 g/L tryptone, 2.5 g/L yeast extract, 5 g/L NaCl, 18.5 g/L brain heart infusion and 91 g/L sorbitol, pH 7.0). The final concentration of chloramphenicol was 30 mg/L for *E. coli* and 20 mg/L for *C. glutamicum.* Isopropyl β-d-Thiogalactoside (IPTG) with a final concentration of 1 mmol/L was added when the OD_600_ of the cells reached about 0.5.

### DNA manipulation and Plasmid construction

*Corynebacterium glutamicum* genomic DNA was isolated using a genomic isolation kit (CWBIO, China). Kits for plasmid isolation, DNA gel extraction, and PCR product purification were purchased from Axygen (China). PCR was carried out using PrimerSTAR (TaKaRa, China). T4 ligase and restriction enzymes were purchased from New England Biolabs (Ipswich, MA, USA). All the DNA manipulation procedures, including PCR, restriction enzyme digestion, ligation, and agarose gel electrophoresis were carried out following the standard procedures. After successfully constructed in *E. coli* DH5α, each plasmid was transformed into *C. glutamicum* CGMCC1.15647 by electroporation as previously described.

**Fig. 6:**
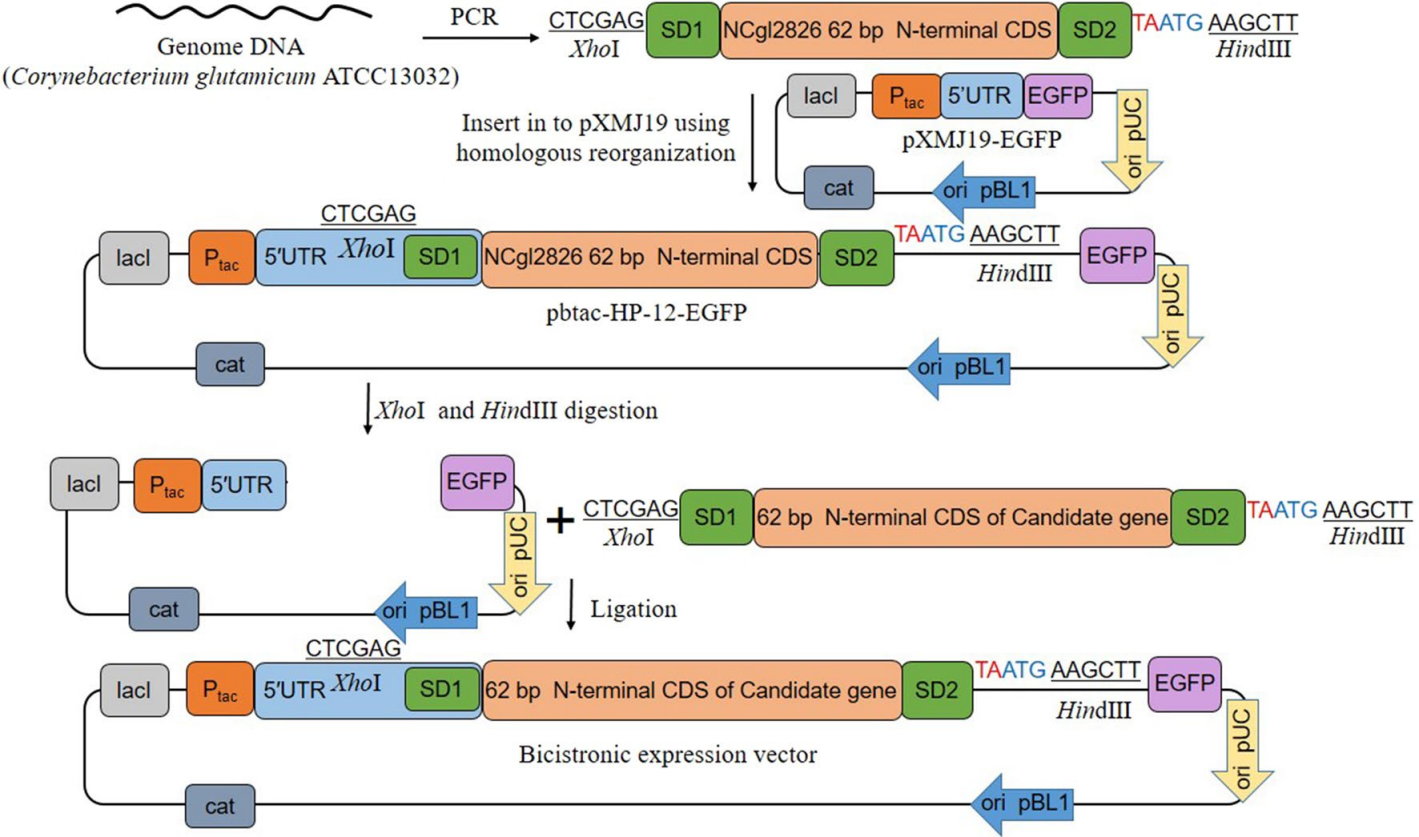
Construction process of enhanced expression plasmids. The 62 bp of the N-terminal coding sequences of 24 genes were constructed into the pXMJ19-EGFP according to this bicistronic expression model and followed a conserved SD2 sequence AAAGGAGGACAACTA

All the primers used in this study were listed in Additional file 1: Table S2. To obtain the expression vectors harboring different fore-cistronic sequences, a pioneering bicistronic plasmid pbtac-HP-12 was constructed as follows: first, the 62 bp of the N-terminal coding sequence of the candidate gene, *NCgl2826*, was amplified from *C. glutamicum* ATCC13032 genome; Primers conferred an *Xho*I cleavage site and a conserved SD sequence (terms as SD1) at the 5′ end of the PCR product. Another SD sequence (terms as SD2) with a translation coupling frame (TAATG) was introduced at the 3′ end of the sequence. Then the above fore-cistronic amplicon was inserted upstream of the MCS region of the HindIII digested original vector pXMJ19-EGFP by the homologous recombination kit (Vazyme, China) (Fig. 6).

The remaining 23 bicistronic expression plasmids were constructed by substituting the fore-cistron region in pbtac-HP-12 with the corresponding 23 fore-cistron fragments amplified from *C. glutamicum* ATCC13032. Here the previously added cleavage site *Xho*I at the N-terminal of fore-cistron was paired with HindIII for fragments insertion.

To compare the strength of these enhanced expression vectors, PCR products of EGFP were digested with HindIII/EcoRI and then inserted into all the 24 bicistronic expression plasmids. Meanwhile, the other *EGFP* PCR fragments were digested with Xho1/EcoRI and then ligated into pbtac-HP-12 to obtain a monocistronic control pXMJ19-EGFP. To further test the expression ability of the top three strongest bicistronic vectors, PCR products of *ADH* (GenBank: AGN20313.1), *RamA* (GenBank: AGN20080.1), *GD* (GenBank: MH370856.1), *BoIFN*-*α* (GenBank: EU276064.1), *PΙNP* (GenBank: X00820.1) (flanked with *Hin*dIII/*Eco*RI adaptors) and *ALDH* (GenBank: AGN20304.1) (flanked with *Hin*dIII/*Bam*H1 adaptors) were cloning to these three plasmids, respectively. All the six genes were appended with a 6× histidine tag at the C-terminal during the PCR process. *ADH*, *ALDH* and *RamA* were originated from *C. glutamicum* CGMCC1.15647, while the codon-optimized *GD* (GenBank: MN.816264), *BoIFN*-*α* (GenBank: MN.816265) and *PΙNP* (contained a cspB signal peptide) (GenBank: MN.816263) genes were synthesized by the company of Shenggong (Shanghai, China). To obtain the monocistronic control vectors expressing the above proteins, each encoding gene was individually amplified again and ligated to pbtac-HP-12. The cloning site here was *Xho*1/*Bam*H1 for *ALDH* and *Xho*1/*Eco*RI for the rest.

### GFP intensity measurement

Cells harboring EGFP plasmids were diluted to a moderate concentration (OD_600_ ≈ 0.5) and the total fluorescence intensity was measured by a fluorescence spectrophotometer (the excitation wavelength: 488 nm, the emission wavelength: 507 nm). The unit fluorescence intensity of each sample was calculated through normalizing the total fluorescence intensity by OD_600_. The EGFP expression level was also analyzed by sodium dodecyl sulfate-polyacrylamide gel electrophoresis (SDS-PAGE).

### Real-time quantitative PCR (qPCR) and calculation of relative translation efficiency

To analyze the transcriptional level of EGFP, strains were grown in 10 mL LBB medium for 24 h, harvested by centrifugation and washed twice with ice-cooled PBS. Total RNA extraction, reverse transcription, and qPCR were then performed using kits from TaKaRa (Dalian, China) according to the manufacturer’s instructions. The PCR condition was: 95 °C for 30 s and 45 cycles at 95 °C for 15 s, 62 °C for 30 s, 72 °C for 20 s. The relative EGFP transcription level was analyzed by the 2^−ΔΔCt^ method and the transcript level of housekeeping gene *16S* rRNA was used as the endogenous control. The EGFP transcription level of pXMJ19-EGFP with a monocistronic P_tac_ was defined as 1. Every sample was measured across at least three biological repeats, which had three duplicated wells each. The translation efficiency is calculated through dividing the EGFP intensity by mRNA abundance, the EGFP translation efficiency (T) = the EGFP expression level (P)/the relative mRNA abundance (M) [25]. The translation efficiency of P_tac_ control here are defined as 1.

### Protein preparation and western blotting assay

After 24 h of cultivation, the cells were harvested by centrifugation at 12,000*g* for 5 min at 4 °C, washed twice with PBS, and then disrupted by sonication on ice. For soluble expressed proteins EGFP, ADH, ALDH, RamA and gD, the lysates were centrifuged at 12,000*g* for 15 min and the supernatants were collected for the subsequent protein analysis. For proteins expressed mainly in the inclusion form (BoIFN-α), the whole lysates were used directly. For protein PΙNP, just take the medium supernatant to further analysis. All the protein samples were analyzed by 12% (w/v) SDS-PAGE.

The proteins on the SDS-PAGE gel were electrophoretically transferred onto a polyvinyl difluoride membrane using a Bio-Rad transblot device (USA). The membrane was incubated within 5% non-fat milk powder for 2 h to block nonspecific binding sites. After incubation, replaced the milk with monoclonal horseradish peroxidase (HRP)-conjugated anti-His_6_ antibody. Washed the membrane three times with TBST after 1 h of incubation. Finally, the protein was performed using an ECL kit (Amersham Biosciences, America). The strength of protein bands was quantified using Image J software.

### Fed-batch cultivation and purification of PΙNP

To validate large-scale expression of EGFP of the top three strongest vectors and achieve large-scale production of PΙNP. After overnight activation, 200 mL of *C. glutamicum* seed solutions were all transferred to 1.8 L of LBB medium (30 g/L glucose) in a 5 L fermenter (Applikon EZ-control). Throughout the total 48 h of cultivation, the temperature was maintained at 30 °C. The dissolved oxygen was maintained at 30% (v/v). The speed was set to 400–1000 r/min and the pH of the medium was controlled at 7. To avoid glucose starvation, 50 mL glucose solution (300 g/L) was added every 4 h after 12 h of inoculation. Glucose concentrations in the culture medium were monitored by a glucose assay kit (Sigma, St. Louis, Missouri, USA).

The protein purification steps were presented below: the medium supernatant contained PΙNP were collected first, protein purification used an AKTA purifier system (GE, Sweden) and a HisTrap HP affinity column. Protein quantity and purity was determined by SDS-PAGE analysis.

## Supplementary information

**Additional file 1.** The bacteria strains, plasmids, primers and gene sequences used in this study.

## Data Availability

The datasets and material used during this study are available from the corresponding author.

## Abbreviations

5′UTR: 5′untranslated region
ADH: Alcohol dehydrogenase
ALDH: Aldehyde dehydrogenase
BCD: Bicistronic design
BoIFN-α: Bovine interferon-α
CDS: Coding sequence
*E. coli*: *Escherichia coli*
EGFP: Enhanced green fluorescent protein
gD: Glycoprotein D protein
IBRV: Infectious bovine rhinotracheitis virus
NT-proBNP: N-terminal pro-brain natriuretic peptide
ORF: Open reading frame
PΙNP: Procollagen type Ι N-terminal peptide
P_tac_: Tac promoter
qPCR: Real-time quantitative PCR
RamA: Regulator of acetate metabolism
scFv: Single-chain variable fragment
SDS-PAGE: Sodium dodecyl sulfate-polyacrylamide gel electrophoresis
TIR: Translation initiation region

## References

[1] Hasegawa S, Suda M, Uematsu K, Natsuma Y, Hiraga K, Jojima T, Inui M, Yukawa H (2013). Engineering of *Corynebacterium glutamicum* for high-yield l-valine production under oxygen deprivation conditions. Appl Environ Microbiol.

[2] Yim SS, An SJ, Kang M, Lee J, Jeong KJ (2013). Isolation of fully synthetic promoters for high-level gene expression in *Corynebacterium glutamicum*. Biotechnol Bioeng.

[3] Becker J, Zelder O, Häfner S, Schroder H, Wittmann C (2011). From zero to hero—design-based systems metabolic engineering of *Corynebacterium glutamicum* for l-lysine production. Metab Eng.

[4] Liu X, Yang Y, Zhang W, Sun Y, Peng F, Jeffrey L, Harvey L, McNeil B, Bai Z (2016). Expression of recombinant protein using *Corynebacterium glutamicum*: progress, challenges and applications. Crit Rev Biotechnol.

[5] Yim SS, An SJ, Choi JW, Ryu AJ, Jeong KJ (2014). High-level secretory production of recombinant single-chain variable fragment (scFv) in *Corynebacterium glutamicum*. Appl Microbiol Biotechnol.

[6] Peng F, Liu X, Wang X, Chen J, Liu M, Yang Y, Bai Z (2019). Triple deletion of clpC, porB, and mepA enhances production of small ubiquitin-like modifier-N-terminal pro-brain natriuretic peptide in *Corynebacterium glutamicum*. J Ind Microbiol Biotechnol.

[7] Srivastava P, Deb JK (2005). Gene expression systems in corynebacteria. Protein Express Purif..

[8] Zhang W, Yang Y, Liu X, Liu C, Bai Z (2019). Development of a secretory expression system with high compatibility between expression elements and an optimized host for endoxylanase production in *Corynebacterium glutamicum*. Microb Cell Fact.

[9] Matsuda Y, Itaya H, Kitahara Y, Theresia N, Kutukova E, Yomantas YA, Date M, Kikuchi Y, Wachi M (2014). Double mutation of cell wall proteins CspB and PBP1a increases secretion of the antibody Fab fragment from *Corynebacterium glutamicum*. Microb Cell Fact.

[10] Tateno T, Fukuda H, Kondo A (2007). Direct production of l-lysine from raw corn starch by *Corynebacterium glutamicum* secreting Streptococcus bovis alpha-amylase using cspB promoter and signal sequence. Appl Microbiol Biotechnol.

[11] Shang X, Chai X, Lu X, Li Y, Zhang Y, Wang G, Zhang C, Liu S, Zhang Y, Ma J (2017). Native promoters of *Corynebacterium glutamicum* and its application in l-lysine production. Biotechnol Lett.

[12] Okibe N, Suzuki N, Inui M, Yukawa H (2009). Isolation, evaluation and use of two strong, carbon source-inducible promoters from *Corynebacterium glutamicum*. Lett Appl Microbiol.

[13] Kim MJ, Yim SS, Choi JW, Jeong KJ (2016). Development of a potential stationary-phase specific gene expression system by engineering of SigB-dependent cg3141 promoter in *Corynebacterium glutamicum*. Appl Microbiol Biotechnol.

[14] Wei L, Xu N, Wang Y, Zhou W, Han G, Ma Y, Liu J (2018). Promoter library-based module combination (PLMC) technology for optimization of threonine biosynthesis in *Corynebacterium glutamicum*. Appl Microbiol Biotechnol.

[15] Rytter JV, Helmark SR, Chen J, Lezyk MJ, Solem C, Jensen PR (2014). Synthetic promoter libraries for *Corynebacterium glutamicum*. Appl Microbiol Biotechnol..

[16] Zhang S, Liu D, Mao Z, Mao Y, Ma H, Chen T, Zhao X, Wang Z (2018). Model-based reconstruction of synthetic promoter library in *Corynebacterium glutamicum*. Biotechnol Lett.

[17] Seo SW, Yang J-S, Kim I, Yang J, Min BE, Kim S, Jung GY (2013). Predictive design of mRNA translation initiation region to control prokaryotic translation efficiency. Metab Eng.

[18] Shi F, Luan M, Li Y (2018). Ribosomal binding site sequences and promoters for expressing glutamate decarboxylase and producing gamma-aminobutyrate in *Corynebacterium glutamicum*. AMB Express..

[19] Jang SH, Cha JW, Han NS, Jeong KJ (2018). Development of bicistronic expression system for the enhanced and reliable production of recombinant proteins in *Leuconostoc citreum*. Sci Rep..

[20] Zhang B, Zhou N, Liu Y, Liu C, Lou C, Jiang C, Liu S (2015). Ribosome binding site libraries and pathway modules for shikimic acid synthesis with *Corynebacterium glutamicum*. Microb Cell Fact.

[21] Mutalik VK, Guimaraes JC, Cambray G, Lam C, Endy D (2013). Precise and reliable gene expression via standard transcription and translation initiation elements. Nat Methods.

[22] Kimura S, Umemura T, Iyanagi T (2005). Two-cistronic expression plasmids for high-level gene expression in *Escherichia coli* preventing translational initiation inhibition caused by the intramolecular local secondary structure of mRNA. J Biochem.

[23] Claassens NJ, Finger-Bou M, Scholten B, Muis F, de Groot JJ, de Gier JW, de Vos WM, van der Oost J (2019). Bicistronic design-based continuous and high-level membrane protein production in *Escherichia coli*. ACS Synth Biol..

[24] Jang SA, Sung BH, Cho JH, Kim SC (2009). Direct expression of antimicrobial peptides in an intact form by a translationally coupled two-cistron expression system. Appl Environ Microbiol.

[25] Zhao Z, Liu X, Zhang W, Yang Y, Dai X, Bai Z (2016). Construction of genetic parts from the *Corynebacterium glutamicum* genome with high expression activities. Biotechnol Lett.

[26] Liu XX, Zhao ZH, Zhang W, Sun Y, Yang YK, Bai ZH (2017). Bicistronic expression strategy for high-level expression of recombinant proteins in *Corynebacterium glutamicum*. Eng Life Sci.

[27] Osadska M, Bonkova H, Krahulec J, Stuchlik S, Turna J (2014). Optimization of expression of untagged and histidine-tagged human recombinant thrombin precursors in *Escherichia coli*. Appl Microbiol Biotechnol.

[28] Arndt A, Eikmanns BJ (2007). The alcohol dehydrogenase gene adhA in *Corynebacterium glutamicum* is subject to carbon catabolite repression. J Bacteriol.

[29] Shah A, Blombach B, Gauttam R, Eikmanns BJ (2018). The RamA regulon: complex regulatory interactions in relation to central metabolism in *Corynebacterium glutamicum*. Appl Microbiol Biotechnol.

[30] Subhadra B, Lee JK (2013). Elucidation of the regulation of ethanol catabolic genes and ptsG using a glxR and adenylate cyclase gene (cyaB) deletion mutants of *Corynebacterium glutamicum* ATCC 13032. J Microbiol Biotechnol.

[31] Shi X, Xia C, Pan B, Wang M (2006). Interferon-alpha genes from Bos and *Bubalus bubalus*. Anim Biotechnol..

[32] Abdelmagid OY, Minocha HC, Collins JK, Chowdhury SI (1995). Fine mapping of bovine herpesvirus-1 (BHV-1) glycoprotein-D (gD) neutralizing epitopes by type-specific monoclonal-antibodies and sequence comparison with BHV-5 gD. Virology.

[33] Veidal SS, Vassiliadis E, Bay-Jensen A-C, Tougas G, Vainer B, Karsdal MA (2010). Procollagen type I N-terminal propeptide (PINP) is a marker for fibrogenesis in bile duct ligation-induced fibrosis in rats. Fibrogenesis Tissue Repair..

[34] Freudl R (2017). Beyond amino acids: use of the *Corynebacterium glutamicum* cell factory for the secretion of heterologous proteins. J Biotechnol.

[35] Tsuchiya M, Morinaga Y (1988). Genetic control systems of *Escherichia coli* can confer inducible expression of cloned genes in coryneform bacteria. Nat Biotechnol.

[36] Mukhopadhyay UK, Sahni G (2002). An insight into the possible mechanism of working of two-cistronic gene expression systems and rational designing of newer systems. J Biosci.

[37] Goodman DB, Church GM, Kosuri S (2013). Causes and effects of N-terminal codon bias in bacterial genes. Science.

[38] Levin-Karp A, Barenholz U, Bareia T, Dayagi M, Zelcbuch L, Antonovsky N, Noor E, Milo R (2013). Quantifying translational coupling in *E. coli* synthetic operons using RBS modulation and fluorescent reporters. ACS Synth Biol..

[39] Islam RS, Tisi D, Levy MS, Lye GJ (2007). Framework for the rapid optimization of soluble protein expression in *Escherichia coli* combining microscale experiments and statistical experimental design. Biotechnol Prog.

[40] Shao J, Cao C, Bao J, Liu H, Peng T, Gao M, Wang J (2015). Characterization of bovine interferon alpha1: expression in yeast *Pichia pastoris*, biological activities, and physicochemical characteristics. J Interferon Cytokine Res.

