# Supplementary material for: Enhanced production of recombinant proteins in Corynebacterium glutamicum by constructing a bicistronic gene expression system
Source: Microb Cell Fact. 2020 May 26;19:113. doi: 10.1186/s12934-020-01370-9 (PMC7251831; doi:10.1186/s12934-020-01370-9)
Supplement: Supplementary file 1 — Additional file 1. The bacteria strains, plasmids, primers and gene sequences used in this study. [file 12934_2020_1370_MOESM1_ESM.docx]

***Microbial Cell Factories***

Additional Materials

**Enhanced production of recombinant proteins in *Corynebacterium glutamicum* by constructing a bicistronic gene expression system**

Manman SUN, Xiong GAO, Zihao ZHAO, An LI, Yali WANG, Yankun, YANG, Xiuxia LIU*, Zhonghu BAI*

National Engineering Laboratory of Cereal Fermentation Technology, Jiangnan University, Wuxi 214112, China

* Correspondence:

Fax: 0510-85320205

E-mail address: liuxiuxia@jiangnan.edu.cn; baizhonghu@jiangnan.edu.cn

**Additional materials**

**Table S1** Bacteria strains and plasmids used in this study

| **Strains or plasmids** | **Description** | **Sources** |
| --- | --- | --- |
| **Strains** |  |  |
| *E.coli* DH5α |  | Lab stock |
| *C. glutamicum* CGMCC1.15647 | Wild type | Lab stock |
| *C. glutamicum* ATCC13032 | Wild type | Lab stock |
| **Plasmids** |  |  |
| pXMJ19 (pXMJ19-0) | *E.coli -C. glutamicum* shuttle vector, chloramphenicol resistance | GenBank: AJ133195.1 |
| pXMJ19-EGFP | pXMJ19, carrying EGFP gene | This study |
| pXMJ19-ALDH | pXMJ19, carrying ALDH gene | This study |
| pXMJ19-ADH | pXMJ19, carrying ADH gene | This study |
| pXMJ19-RamA | pXMJ19, carrying RamA gene | This study |

**Table S2** Primers in this study

| **Primer** | **Sequence (5′-3′)^a^** | **Intension** |
| --- | --- | --- |
| HT-1-F | CCG **CTCGAG** AAAGGAGGACAACC  ATGGCTCTTACTTCTGAGCA GAAGAAGTC | Amplifying HT-1 |
| HT-1-R | CCC **AAGCTT** CAT TAGTTGTCCTCCTTT  TCGGTCTCGTGGAGGCCG | Amplifying HT-1 |
| HT-2-F | CCG **CTCGAG** AAAGGAGGACAACC  ATGGCTACCACAGCTTCCAAGATCTC | Amplifying HT-2 |
| HT-2-R | CCC **AAGCTT** CAT TAGTTGTCCTCCTTT  CTCCAAAGAGCATCTTGCTGTGCTG | Amplifying HT-2 |
| HT-3-F | CCG **CTCGAG** AAAGGAGGACAACC  GTGGCAAAGGCGAAGTTCGAGC | Amplifying HT-3 |
| HT-3-R | CCC **AAGCTT** CAT TAGTTGTCCTCCTTT  ACGTGACCGATGGTGCCGATG | Amplifying HT-3 |
| HT-4-F | CCG **CTCGAG** AAAGGAGGACAACC  ATGGCAAACCCAAGAAACGAAGCA | Amplifying HT-4 |
| HT-4-R | CCC **AAGCTT** CAT TAGTTGTCCTCCTTT  TCGGTCTCAGCGAAACGTGC | Amplifying HT-4 |
| HT-5-F | CCG **CTCGAG** AAAGGAGGACAACC  GTGGGAGATGTTGTAAAAGGCAACGAC | Amplifying HT-5 |
| HT-5-R | CCC **AAGCTT** CAT TAGTTGTCCTCCTTT  ATTTTTCGGCGCGTATCACCGTC | Amplifying HT-5 |
| HT-6-F | CCG **CTCGAG** AAAGGAGGACAACC  ATGGGACGTGCAGTAGGAATTGACC | Amplifying HT-6 |
| HT-6-R | CCC **AAGCTT** CAT TAGTTGTCCTCCTTT  CCTTCAAGTACGGAAACCACAGAGTTG | Amplifying HT-6 |
| HT-7-F | CCG **CTCGAG** AAAGGAGGACAACC  ATGACGAACAATGCATTAACCGTGC | Amplifying HT-7 |
| HT-7-R | CCC **AAGCTT** CAT TAGTTGTCCTCCTTT  GCCGTGAGCAAATCCGCCT | Amplifying HT-7 |
| HT-8-F | CCG **CTCGAG** AAAGGAGGACAACC  ATGTACGCGATCGTCAAGACCG | Amplifying HT-8 |
| HT-8-R | CCC **AAGCTT** CAT TAGTTGTCCTCCTTT  TTAACGAGGTCACCTTCGGCAACC | Amplifying HT-8 |
| HT-9-F | CCG **CTCGAG** AAAGGAGGACAACC  GTGAAGAGTTCTGTCGAGAAGCTGAG | Amplifying HT-9 |
| HT-9-R | CCC **AAGCTT** CAT TAGTTGTCCTCCTTT  AATGGAACCTCAACGGTGATCTTTGAAC | Amplifying HT-9 |
| HT-10-F | CCG **CTCGAG** AAAGGAGGACAACC  GTGGCTGGTTCCTCCCACAC | Amplifying HT-10 |
| HT-10-R | CCC **AAGCTT** CAT TAGTTGTCCTCCTTT  TCATCAAGGGTGGATACACCGCG | Amplifying HT-10 |
| HT-11-F | CCG **CTCGAG** AAAGGAGGACAACC  ATGACTCGACGTCTACATGGTGGTG | Amplifying HT-11 |
| HT-11-R | CCC **AAGCTT** CAT TAGTTGTCCTCCTTT  TTTAGCTGTCCTTTAACGTGTTCCTGGC | Amplifying HT-11 |
| HT-12-F | CCG **CTCGAG** AAAGGAGGACAACC  GTGTCGCTTTCAGAGCAGGAGC | Amplifying HT-12 |
| HT-12-R | CCC **AAGCTT** CAT TAGTTGTCCTCCTTT  TCTGCCATGAGCGCTTGCTC | Amplifying HT-12 |
| HP-1-F | CCG **CTCGAG** AAAGGAGGACAACC  GTGGCAAACGTCAACATCAAGCCG | Amplifying HP-1 |
| HP-1-R | CCC **AAGCTT** CAT TAGTTGTCCTCCTTT  TCTGCTTCGTTGATCTGAACGAGGATC | Amplifying HP-1 |
| HP-2-F | CCG **CTCGAG** AAAGGAGGACAACC  ATGACTGAACGTACTCTCAT CCTTATCAAGC | Amplifying HP-2 |
| HP-2-R | CCC **AAGCTT** CAT TAGTTGTCCTCCTTT  TCGCCGACGTGTCCGTTG | Amplifying HP-2 |
| HP-3-F | CCG **CTCGAG** AAAGGAGGACAACC  ATGCCTATCGCAACTCCCGAGG | Amplifying HP-3 |
| HP-3-R | CCC **AAGCTT** CAT TAGTTGTCCTCCTTT  AATCCGCCTTCCTTAGCACGATC | Amplifying HP-3 |
| HP-4-F | CCG **CTCGAG** AAAGGAGGACAACC  ATGGGGTCCATGGCTAAAACACATTTTC | Amplifying HP-4 |
| HP-4-R | CCC **AAGCTT** CAT TAGTTGTCCTCCTTT  GGCAGTTCGCCGGAGGTAG | Amplifying HP-4 |
| HP-5-F | CCG **CTCGAG** AAAGGAGGACAACC  ATGAGCGAGAATTACAGCAAGATTGTCGTT | Amplifying HP-5 |
| HP-5-R | CCC **AAGCTT** CAT TAGTTGTCCTCCTTT  GCTAGAAGGGACGACTTAGATCCATCAG | Amplifying HP-5 |
| HP-6-F | CCG **CTCGAG** AAAGGAGGACAACC  GTGGCCCGTGTAGTTGTCAATGTC | Amplifying HP-6 |
| HP-6-R | CCC **AAGCTT** CAT TAGTTGTCCTCCTTT  GCCTGCCCCTGGGGATC | Amplifying HP-6 |
| HP-7-F | CCG **CTCGAG** AAAGGAGGACAACC  ATGACTAACGGAAAATTGATTCTTCTTCGTCACG | Amplifying HP-7 |
| HP-7-R | CCC **AAGCTT** CAT TAGTTGTCCTCCTTT  TGGTTGGATGCGTTCCATTCGC | Amplifying HP-7 |
| HP-8-F | CCG **CTCGAG** AAAGGAGGACAACC  GTGGCTGAAATCATGCACGTATTCG | Amplifying HP-8 |
| HP-8-R | CCC **AAGCTT** CAT TAGTTGTCCTCCTTT  ACGGTTGGGTTACCGCGG | Amplifying HP-8 |
| HP-9-F | CCG **CTCGAG** AAAGGAGGACAACC  ATGAGCGATATTCGTATGGCAGCCC | Amplifying HP-9 |
| HP-9-R | CCC **AAGCTT** CAT TAGTTGTCCTCCTTT  CGATCAAAGACGTCATTTCCGAAACCAG | Amplifying HP-9 |
| HP-10-F | CCG **CTCGAG** AAAGGAGGACAACC  ATGCGACTCGTACTCCTCGGAC | Amplifying HP-10 |
| HP-10-R | CCC **AAGCTT** CAT TAGTTGTCCTCCTTT  GAGAGAATTGCAGCCTGGGTGC | Amplifying HP-10 |
| HP-11-F | CCG **CTCGAG** AAAGGAGGACAACC  ATGATTGGAGCACCACCCGACAT | Amplifying HP-11 |
| HP-11-R | CCC **AAGCTT** CAT TAGTTGTCCTCCTTT  TGGCCGATGGTTTCGGTGATG | Amplifying HP-11 |
| HP-12-F | CCTGCGCTTCTAGTGCAATT**CTCGAG**AAAGGAGGACAACC  ATGGCTGTATACGAACTCCCAGAACTC | Amplifying HP-12 |
| HP-12-R | GACCTGCAGGCATGC**AAGCTT** CATTAGTTGTCCTCCTTT  GCGATGTGTGGCTCGAGAGC | Amplifying HP-12 |
| EGFP-F1 | CCG **CTCGAG** AAAGGAGGACAACTA ATG **AAGCTT**  ATGGTGAGCA AGGGCGAGGA | Amplifying EGFP fragment for pXMJ19 |
| EGFP-F2 | CCC **AAGCTT**  ATGGTGAGCA AGGGCGAGGA | Amplifying EGFP fragment for enhanced expression vectors |
| EGFP-R | CGC **GAATTC**  TTACTTGTACAGCTCGTCCATGCCG | Amplifying EGFP |
| ALDH-F1 | CCG **CTCGAG** AAAGGAGGACAACTA **AAGCTT**  ATGACTGTCT ACGCAAATCC AGGAACC | Amplifying ALDH fragment for pXMJ19 |
| ALDH-F2 | CCC **AAGCTT**  ATGACTGTCT ACGCAAATCC AGGAACC | Amplifying ALDH fragment for enhanced expression vectors |
| ALDH-R | CGC **GGATCC** TTAGTGGTGATGGTGGTGATG  GAACAGTCCGGTTGGGTTATCGTAG | Amplifying ALDH |
| ADH-F1 | CCG **CTCGAG** AAAGGAGGACAACTA **AAGCTT**  ATGACCACTG CTGCACCCC | Amplifying ADH fragment for pXMJ19 |
| ADH-F2 | CCC **AAGCTT**  ATGACCACTG CTGCACCCC | Amplifying ADH fragment for enhanced expression vectors |
| ADH-R | CGC **GAATTC** TTAGTGGTGATGGTGGTGATG  GAAACGAATCGCCACACGACCAT | Amplifying ADH |
| RamA-F1 | CCG **CTCGAG** AAAGGAGGACAACTA **AAGCTT**  GTGGATACCC AGCGGATTAA AGATGAC | Amplifying RamA fragment for pXMJ19 |
| RamA-F2 | CCC **AAGCTT**  GTGGATACCC AGCGGATTAA AGATGAC | Amplifying RamA fragment for enhanced expression vectors |
| RamA-R | CGC **GAATTC** TTAGTGGTGATGGTGGTGATG  AGGCAGTGCGCCGATCC | Amplifying RamA |
| PΙNP-F1 | CCG **CTCGAG** AAAGGAGGACAACTA **AAGCTT**  ATGTTTA ACAATCGTAT CCGCACTGCA | Amplifying PΙNP fragment for pXMJ19 |
| PΙNP-F2 | CCC **AAGCTT**  ATGTTTA ACAATCGTAT CCGCACTGCA | Amplifying PΙNP fragment for enhanced expression vectors |
| PΙNP –R | CGC **GAATTC** TTAGTGGTGATGGTGGTGATG  GCCCTTATCATCGTCGTCCTTGTAATC | Amplifying PΙNP |
| gD-F1 | CCG **CTCGAG** AAAGGAGGACAACTA **AAGCTT**  ATGCCAATGCCACGCTACAACTACACC | Amplifying gD fragment for pXMJ19 |
| gD -F2 | CCC **AAGCTT**  ATGCCAATGCCACGCTACAACTACACC | Amplifying gD fragment for enhanced expression vectors |
| gD –R | CGC **GAATTC** TTAGTGGTGATGGTGGTGATG  TGCTTCTGGGGTTGGGGATTCA | Amplifying gD |
| BoIFN-α-F1 | CCG **CTCGAG** AAAGGAGGACAACTA **AAGCTT**  ATGTGCCACCTTCCACACACCCA | Amplifying BoIFN-α fragment for pXMJ19 |
| BoIFN-α -F2 | CCC **AAGCTT**  ATGTGCCACCTTCCACACACCCA | Amplifying BoIFN-α fragment for enhanced expression vectors |
| BoIFN-α -R | CGC **GAATTC** TTAGTGGTGATGGTGGTGATG  ATCCTTGCGGCGAAAGGATTCCT | Amplifying BoIFN-α |
| EGFP- qPCR-F | ACAACATCGAGGACGGCAG | qPCR primer for EGFP |
| EGFP-qPCR-R | GTCCATGCCGAGAGTGATCC | qPCR primer for EGFP |
| 16s-qPCR-F | TCGGAGTCGCTAGTAATCGCAGAT | qPCR primer for 16S |
| 16s-qPCR-R | GGCTTCGGGTGTTACCAACTTTCA | qPCR primer for 16S |

^a^ The underlined sequences are homology arms and the sites for the restriction enzymes are in boldface. Green sequences represent SD1 sequences, red sequences ended with TAATG are SD2 sequences in the bicistronic expression pattern and blue sequences represent 6×his tag.

**Table S3** Gene sources information

12 highly transcribed genes (HT-set) and 12 highly expressed genes (HP-set) in *C. glutamate*

| **Gene ID** | **Gene name** | **Number** | **Gene ID** | **Gene name** | **Number** |
| --- | --- | --- | --- | --- | --- |
| NCgl1901 | rpsO | HT-1 | NCgl0572 | groES | HP-1 |
| NCgl2252 | NCgl2252 | HT-2 | NCgl2287 | ndk | HP-2 |
| NCgl0480 | tuf | HT-3 | NCgl2673 | NCgl2673 | HP-3 |
| NCgl0468 | rplJ | HT-4 | NCgl1041 | tpx | HP-4 |
| NCgl1504 | NCgl1504 | HT-5 | NCgl1316 | NCgl1316 | HP-5 |
| NCg2702 | dnaK | HT-6 | NCgl2501 | NCgl2501 | HP-6 |
| NCgl2177 | NCgl2177 | HT-7 | NCgl0390 | gpmA | HP-7 |
| NCgl2280 | rplU | HT-8 | NCgl0935 | eno | HP-8 |
| NCgl2329 | tig | HT-9 | NCgl2328 | clpP | HP-9 |
| NCgl1941 | NCgl1941 | HT-10 | NCgl0533 | adk | HP-10 |
| NCgl0576 | NCgl0576 | HT-11 | NCgl2473 | NCgl2473 | HP-11 |
| NCgl2088 | NCgl2088 | HT-12 | NCgl2826 | NCgl2826 | HP-12 |

**Additional Data 1. Gene sequences**

**Codon-optimized PΙNP+his**

ATGCAAGAAGAAGGCCAAGTGGAAGGTCAAGATGAGGACATCCCACCAATCACTTGCGTGCAGAACGGTCTGCGCTACCACGACCGTGATGTCTGGAAACCAGAACCTTGCCGCATCTGCGTGTGCGACAACGGCAAGGTGCTGTGCGACGACGTGATCTGCGATGAAACCAAGAACTGCCCGGGCGCAGAAGTGCCAGAAGGCGAATGCTGCCCAGTGTGCCCAGATGGCTCCGAGTCCCCAACCGATCAAGAAACCACCGGCGTCGAGGGCCCTAAGGGCGATACTGGTCCTCGTGGTCCACGCGGTCCAGCCGGCCCTCCGGGCCGTGACGGCATCCCGGGCCAGCCGGGCCTCCCGGGCCCACCGGGCCCACCGGGCCCACCGGGCCCACCGGGTCTGGGCGGTAACTTCGCACCAGATTACAAGGACGACGATGATAAGGGCCACCATCATCACCACCATCACCACCATCACCACTAA

**Codon-optimized gD+his**

ATGCCAATGCCACGCTACAACTACACCGAACGCTGGCACACCACCGGCCCAATCCCATCCCCATTCGCAGATGGCCGCGAACAGCCAGTCGAAGTGCGCTACGCTGCATCCGCAGCAGCATGCGATATGCTCGCTCTGATCGCTGATCCACAGGTGGGTCGCACCCTGTGGGAAGCTGTGCGCCGCCACGCACGCGCTTACAACGCAACCGTGATCTGGTACAAGATCGAGTCCGGCTGCGCACGCCCACTCTACTACATGGAATACACCGAATGCGAACCACGCAAGCACTTCGGCTACTGCCGCTACCGTACCCCACCATTCTGGGATTCCTTCCTGGCTGGCTTCGCATACCCAACCGATGATGAGCTGGGCCTGATCATGGCTGCTCCAGCACGCCTGGTGGAAGGCCAGTACCGCCGCGCACTGTACATCGATGGCACCGTGGCTTACACCGATTTCATGGTGTCCCTGCCAGCTGGCGATTGCTGGTTCTCCAAGCTGGGCGCAGCACGCGGCTACACCTTCTCCGCATGCTTCCCTGCACGCGAGTACGAACAGAACAAGGTGCTGCGCCTGACCTACCTGACCCAGTACTACCCACAGGAAGCACACAAGGCTATCGTCGATTACTGGTTCATGCGCCACGGCGGCGTTGTTCCTCCATACTTCGAAGAATCTAAGGGTTACGAACCACCACCAGCTGCTGATGGTGGTTCCCCAGCACCACCAGGTGATGATGAAGCTCGTGAAGATGAAGGTGAAACCGAAGATGGTGCAGCAGGTCGTGAAGGTAACGGTGGCCCACCAGGCCCTGAAGGTGATGGTGAATCCCCAACCCCAGAAGCACATCACCACCATCACCACTAA

**Codon-optimized BoIFN-α+his**

ATGTGCCACCTTCCACACACCCACTCTCTGGCTAACAGACGCGTCCTTATGCTTCTTCAACAGCTGCGCCGCGTTTCCCCATCCTCCTGCCTCCAGGATCGCAACGATTTCGAGTTCTTGCAGGAAGCACTGGGCGGCTCCCAGCTCCAGAAGGCACAGGCAATTTCCGTGCTGCACGAAGTGACACAGCACACCTTCCAACTGTTCTCCACCGAAGGCTCCCCAGCAACCTGGGATAAGTCCCTGCTGGACAAACTGCGCGCAGCACTTGATCAGCAGCTGACCGATCTCCAGGCATGCCTGACCCAGGAAGAAGGCCTGCGCGGCGCACCACTGCTGAAGGAAGATTCCTCCCTGGCAGTGCGCAAGTACTTCCACCGCCTGACATTGTACCTCCAAGAAAAGCGCCACTCCCCATGCGCATGGGAAGTGGTGCGCGCAGAAGTGATGCGCGCATTCTCCTCCTCCACCAACCTCCAGGAATCCTTTCGCCGCAAGGATCATCACCACCATCACCACTAA
